# Long-term survival after repeated resection for lung metastasis originating from pancreatic cancer: a case report

**DOI:** 10.1186/s40792-020-00832-x

**Published:** 2020-04-07

**Authors:** Yasunori Uesato, Koichi Tamashiro, Mitsuhisa Takatsuki

**Affiliations:** 1 Department of Digestive and General Surgery, University of Ryukyus, 207 Uehara, Nishihara, Okinawa, Japan; 2 Department of Pathology, University of Ryukyus, 207 Uehara, Nishihara, Okinawa, Japan

**Keywords:** Pancreatic cancer, Repeated resection of lung metastasis, Prognosis, Long-term survival

## Abstract

**Background:**

Pancreatic cancer has a grave prognosis. Most patients with metastatic pancreatic cancer are inoperable, and case reports of resection of lung metastasis from pancreatic cancer are rare. This patient underwent resection of a lung metastasis twice after pancreaticoduodenectomy for pancreatic cancer.

**Case presentation:**

A 75-year-old man with pancreaticoduodenectomy and adjuvant chemotherapy for pancreatic cancer was diagnosed with a lung metastasis 48 months after surgery. Histological findings after thoracoscopic partial resection of the right lung by video-assisted thoracic surgery confirmed the presence of a lung metastasis originating from the pancreatic cancer. The patient refused chemotherapy. A new lung metastasis was detected 84 months following the second surgery (132 months after the pancreaticoduodenectomy). After thoracoscopic partial resection of the left lung by video-assisted thoracic surgery, the histological findings once again confirmed a metastasis that originated from the pancreatic cancer. The patient refused chemotherapy and remained alive and relapse-free after the 10-month follow-up.

**Conclusion:**

Detection and resection of an isolated lung metastasis originating from pancreatic cancer may improve prognosis. Careful follow-up may be warranted to identify patients who might benefit from aggressive local treatment of oligometastasic pancreatic cancer.

## Background

Pancreatic cancer has a very poor prognosis and high mortality. Patients with metastatic pancreatic cancer have a 5-year overall survival of only 2% [1], and less than 5% of patients who receive chemotherapy for advanced pancreatic cancer survive for more than 5 years [2–5]. Distant metastasis of pancreatic cancer mostly occurs in the liver followed by lungs, regional lymph nodes, and the peritoneum [6]. Successful surgical intervention in patients with an isolated lung recurrence of pancreatic cancer has been reported [7–11], but cases involving multiple pulmonary resection of pancreatic cancer metastasis are very rare. This patient underwent two separate lung resections for pancreatic cancer metastasis with good long-term survival.

## Case presentation

**Fig. 1:**
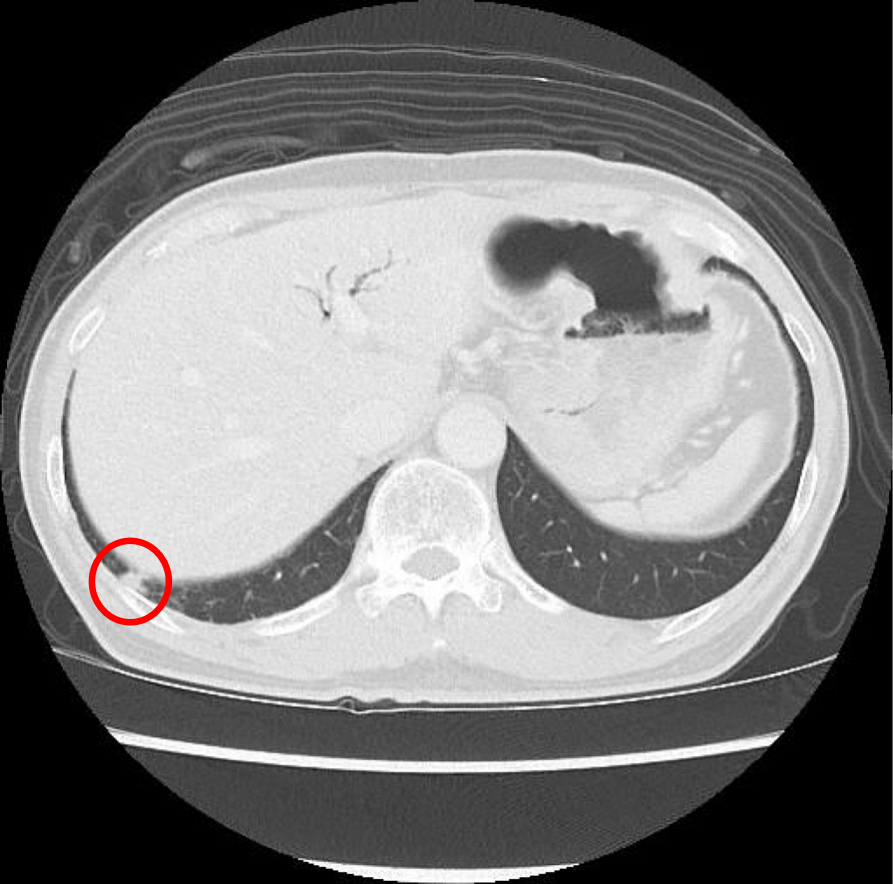
Computed tomography (CT) shows a 5 × 3-mm tumor mass near the base of the right lung. This was a new isolated, localized, solid lesion

**Fig. 2:**
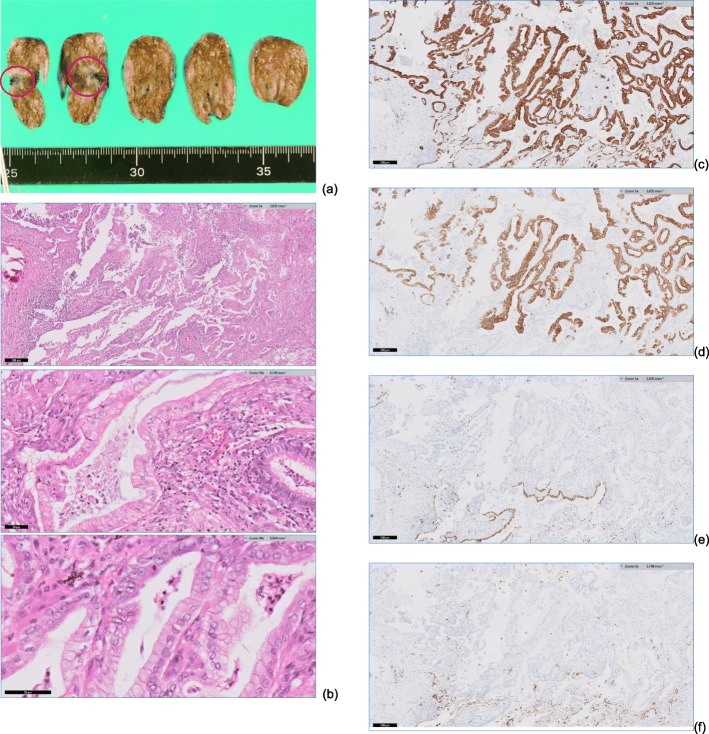
Histopathological and immunostaining findings with **a** the tumor location indicated by the circle and **b** hematoxylin-eosin staining of tissue with columnar tumor cells, nuclear atypia, and mucus containing mucus vesicles. Immunostaining shows **c**, **d** diffuse expression of cytokeratin 7 and 20 and **e**, **f** negative staining of thyroid transcription factor 1 and Napsin A

The patient was a 75-year-old man with a pancreatic tumor that was detected by ultrasonography during a routine medical checkup. Computed tomography (CT) revealed a mass in the head of the pancreas that was diagnosed as pancreatic cancer. Evidence of distant metastasis was not found by CT. The patient’s CEA (1.1 ng/ml) and CA19-9 (8.0 U/ml) were within their normal ranges. The histopathological diagnosis following pancreaticoduodenectomy (PD) with clear margins confirmed a well-differentiated, invasive adenocarcinoma, pT2 (25 × 32 mm), N2, M0, ly3, v2, pStageIII using the eighth edition UICC classification system criteria. Adjuvant chemotherapy with tegafur, gimeracil, and oteracil potassium (100 mg/day) was given for 1 year post-PD. At the 48-month follow-up, a new 5 × 3-mm lesion in the lower right lobe of the lung was detected by CT (Fig. 1). It was a new lesion suggestive of malignant disease, and metastatic lung cancer was suspected because it was an isolated solid mass. The patient’s CEA (1.2 ng/ml) and CA19-9 (5.0 U/ml) concentrations were normal, and other metastases were not detected by CT. Histopathological findings of the lung lesion following thoracoscopic partial resection of the right lung by video-assisted thoracic surgery (VATS) revealed a well-differentiated tumor with irregular growth of atypical gland ducts showing irregular flexion and bifurcation characteristic of a mucus-producing adenocarcinoma. Immunostaining findings were consistent with a lung metastasis of pancreatic cancer rather than a primary lung cancer (Fig. 2). Adjuvant chemotherapy was recommended to prevent recurrence because the lung metastasis was suspected to be pancreatic cancer based on the clinical course, but the patient refused.

**Fig. 3:**
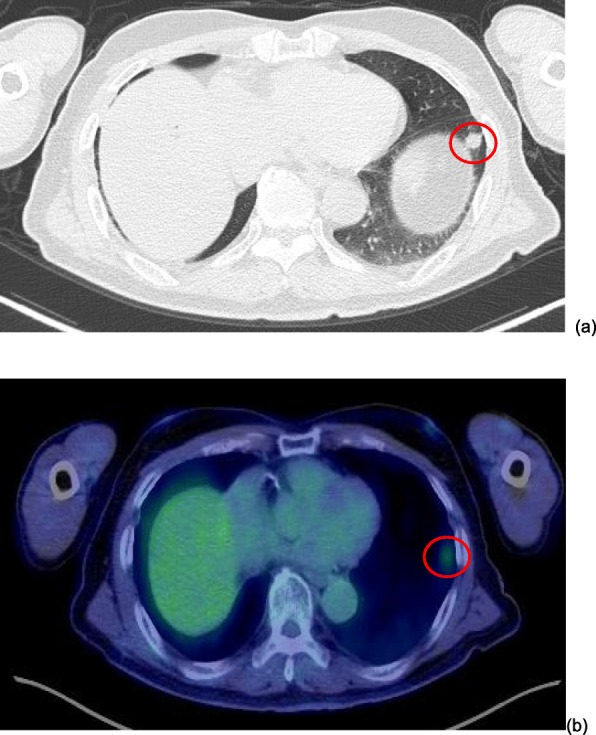
Computed tomography (CT) and positron emission tomography (PET) show a 10 × 10-mm tumor in the left lung S8 (**a**). This was a new isolated, localized, and solid lesion. Lung cancer metastasis was suspected but pleural indentation was also present, suggesting the possibility of primary lung cancer. PET shows strong uptake of fluorodeoxyglucose at the same site, suggesting lung metastasis (**b**)

**Fig. 4:**
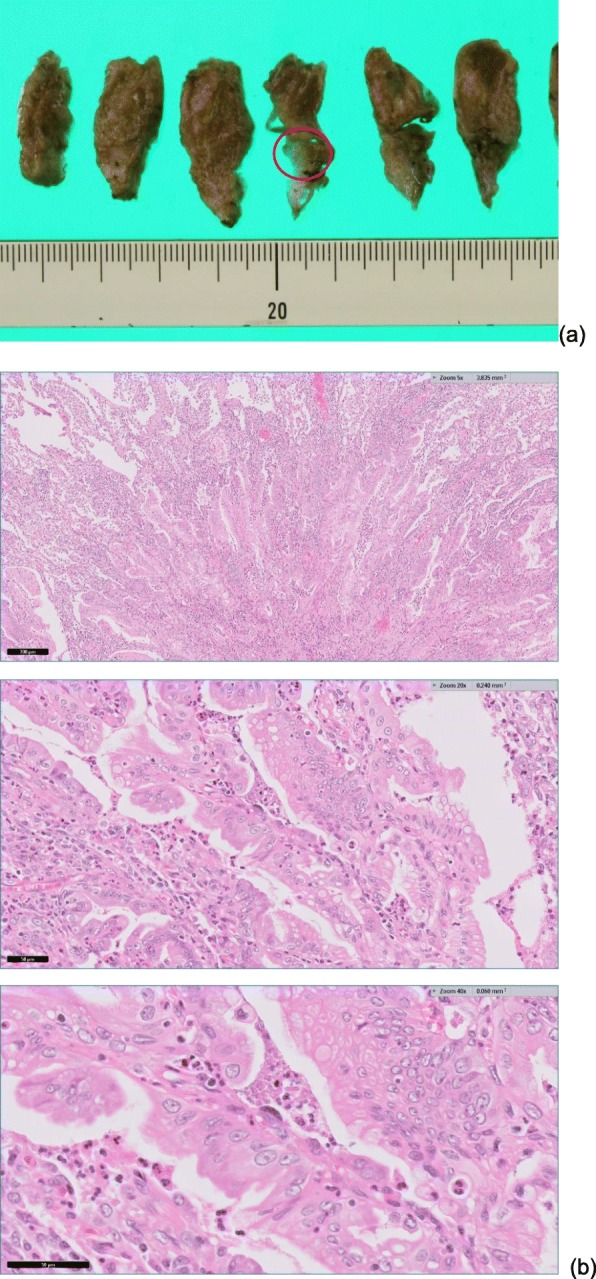
Histopathological findings with **a** the tumor location indicated by the circle and **b** hematoxylin-eosin stained tissue with alveolar replacement and proliferation of atypical gland ducts with irregular mucus-containing branches

Eighty-four months after the first pulmonary resection (132 months after PD), CT and positron emission tomography (PET) revealed a new 10 × 10-mm lesion in the lower lobe of the left lung (Fig. 3). A lung metastasis was suspected because the mass was new, isolated, solid, and had smooth margin. However, pleural invagination was also found, suggesting primary lung cancer. A bronchoscopy for a histopathological diagnosis was not performed because of the tumor location. CT and PET did not find other metastases. Serum CA19-9 was high (90 U/ml, normal < 37 U/ml). VATS was performed and was followed by an uneventful postoperative recovery without severe complications and a return of the CA19-9 concentration to normal levels. The histopathological findings were similar to those for the first lung lesion, finding a well-differentiated adenocarcinoma that was diagnosed as a pancreatic cancer metastasis (Fig. 4). The patient refused adjuvant chemotherapy and had experienced no severe complications or relapse at the 10-month follow-up.

## Discussion

Generally, patients with recurrent pancreatic cancer are indicated for chemotherapy or best supportive care, and not for surgical intervention. The prognosis is extremely poor for recurrence in the liver even if the lesion is solitary [12], but surgical intervention for solitary recurrence in the lung has been reported to improve prognosis [7–11]. Downs-Canner et al. reported that surgery for lung recurrences prolonged overall survival compared with chemotherapy or best supportive care [13]. Our patient remained alive and recurrence-free 142 months after PD, which is far longer than expected for stage III pancreatic cancer.

Previous reports of surgical intervention for pancreatic cancer recurrence in the lung are summarized in Table 1. The mean time to pulmonary resection was 26 months. Most patients had one lung recurrence, and the mean overall survival after lung resection was 34 months. As in this patient, the time to subsequent recurrence was prolonged, and overall survival was improved, by pulmonary resection. Only one previous report included multiple resection procedures, and in line with our experience, the patient also had long-term survival of 8 years after surgery. The available evidence indicated that pulmonary resection for recurrence can significantly prolong survival in selected patients.

**Table 1:** Cases of lung recurrence resection

Author (number of patient)	Age	Gender	Operation method	pStage	Adjuvant chemotherapy	Time until lung recurrence (months)	Number of lung recurrence	OS from lung resection (months)	Histology
Miyasaka et al. (1)	70	Female (1)	PD (1)	IA (1)	GEM/TS-1	16	1 (1)	34	Well differentiated
Kurahara et al. (7)	NA	Men (4), female (3)	NA	NA	NA	NA	1 (7)	37	NA
Ilmer et al. (11)	66 (49–75)	Men (6), female (5)	PD (10), DP (1)	NA	NA	17 (3–64)	1 (11)	26	NA
Tagawa et al. (4)	69.5 (63–82)	Men (2), female (2)	PD (2), DP (2)	IIA (3), IIB (1)	GEM (4)	29 (21–47)	1 (4)	38 (10–65)	NA
Okui et al. (6)	64.5 (63–74)	Men (3), female (3)	PD (3), DP (3)	IB (1), IIA (2), III (1), NA (1)	GEM (4), TS-1 (2)	26 (0–64)	1 (5), 2 (1)	37 (36–48)	Well differentiated (5), mucinous (1)
Kitasato et al. (1)	59	Female (1)	DP (1)	III (1)	5FU + CDDP	163	1 (1)	24 ≤	Well differentiated

*NA* not available, *PD* pancreaticoduodenectomy, *DP* distal pancreatectomy, *GEM* gemcitabine, *TS-1* tegafur/gimeracil/oteracil, *5-FU* 5-fluorouracil, CDDP cisplatin

Distinguishing between primary and metastatic lung cancer is needed to guide treatment. Thyroid transcription factor (TTF)-1 expression has been described as a useful marker of primary lung cancer and highly specific for primary lung adenocarcinoma [14, 15]. TTF-1 immunostaining was negative in this patient. However, because TTF-1 positivity is reported to be low in mucinous bronchioloalveolar carcinoma, it was not conclusive in this case of mucin-producing adenocarcinoma [16]. Cytokeratin (CK)7 and CK20 are useful for distinguishing between lung primary and metastatic tumors. Primary lung cancer has many CK-positive and CK-negative patterns, which are not consistent with the findings of this case, but there are reports of CK7- and-CK20-positive mucinous bronchioloalveolar carcinomas [17, 18]. Napsin A has been reported to be specific for primary lung adenocarcinoma and was negative in this case [19]. Although it was difficult to completely rule out primary lung cancer in this patient by histopathology and immunostaining, the findings were consistent with metastatic lung cancer and were also consistent with the clinical course.

The available evidence of the effectiveness of metastatic resection of pulmonary metastasis of pancreatic cancer is not sufficient to make an overall recommendation. Surgical indications must be carefully considered on a case-by-case basis. The following criteria may help to determine whether surgery is indicated: the patient can tolerate surgery, the primary lesion is controlled, other metastases are not present outside of the lung, and all lung metastases can be resected. This patient and most of the patients in the reports described in Table 1 met those criteria. The overall experience indicates that the survival of patients with a metachronous solitary tumor recurrence and a long disease-free interval following resection of the primary tumor could be prolonged by surgical intervention. This patient met the suggested conditions at the time of the first and second lung resections and experienced prolonged postoperative survival.

It is not clear why the prognosis of patients with metachronous lung recurrence is improved by surgery. Wangjam et al. and Takano et al. suggested that lung recurrences may have a tumor microenvironment different from that of the primary tumor [12, 20]. Kurahara et al. reported that pancreatic cancer cells that form a solitary lung lesion may be less aggressive than those that form multiple lung metastases or metastasize to other organs [11]. A better understanding of the mechanisms underlying differences in metastatic behavior would increase the accuracy of prognosis and help to determine surgical indications for lung recurrence.

## Conclusions

Lung resection twice for metachronous lung metastasis resulted in long-term recurrence-free survival after PD for pancreatic cancer. Rare occurrences of solitary pancreatic cancer metastasis to the lung may be curable with aggressive resection, but the criteria for surgical selection are not clear. Future studies may identify prognostic biomarkers that would help to identify patients who would benefit from surgical intervention, similar to the patient described here.

## Data Availability

Presented within the manuscript. Please contact the author for additional data requests.

## Abbreviations

CT: Computed tomography
PD: Panceraticoduodenectomy
PET: Positron emission tomography
VATS: Video-assisted thoracic surgery
TTF: Transcription factor
CK: Cytokeratin
